# Circadian (De)regulation in Head and Neck Squamous Cell Carcinoma

**DOI:** 10.3390/ijms20112662

**Published:** 2019-05-30

**Authors:** Sadia Rahman, Sandra Kraljević Pavelić, Elitza Markova-Car

**Affiliations:** University of Rijeka, Department of Biotechnology, Centre for High-Throughput Technologies, 51000 Rijeka, Croatia; sadiahusen@gmail.com (S.R.); sandrakp@biotech.uniri.hr (S.K.P.)

**Keywords:** head and neck squamous cell carcinoma (HNSCC), circadian rhythm, circadian clock genes, cancer

## Abstract

Head and neck cancer encompass different malignancies that develop in and around the throat, larynx, nose, sinuses and mouth. Most head and neck cancers are squamous cell carcinomas (HNSCC) that arise in the flat squamous cells that makeup the thin layer of tissue on the surface of anatomical structures in the head and neck. Each year, HNSCC is diagnosed in more than 600,000 people worldwide, with about 50,000 new cases. HNSCC is considered extremely curable if detected early. But the problem remains in treatment of inoperable cases, residues or late stages. Circadian rhythm regulation has a big role in developing various carcinomas, and head and neck tumors are no exception. A number of studies have reported that alteration in clock gene expression is associated with several cancers, including HNSCC. Analyses on circadian clock genes and their association with HNSCC have shown that expression of *PER1*, *PER2, PER3, CRY1, CRY2,* *CKIε, TIM,* and *BMAL1* are deregulated in HNSCC tissues. This review paper comprehensively presents data on deregulation of circadian genes in HNSCC and critically evaluates their potential diagnostics and prognostics role in this type of pathology.

## 1. Introduction

Head and neck cancer are malignant tumors of the throat, larynx, nose, sinuses and mouth. The histological types of head and neck cancer include squamous cell carcinoma, papillary carcinoma, non-Hodgkin lymphoma, basal cell carcinoma, adenocarcinoma, Hodgkin lymphoma and other lymphomas, follicular carcinoma etc. Cancers of the head and neck are more common in men compared to women. The majority of cases, up to 90%, fall into the category of head and neck squamous cell carcinomas (HNSCC). These tumors develop from the flat squamous cells within the epithelium on the surface of head and neck anatomical structures. The major risk factors for HNSCC include, but are not limited to, tobacco use, alcohol consumption, various viral infections, particularly the human papilloma virus 16 (HPV16) infections, genetic polymorphism or damage. The prevalence of HPV in HNSCC is estimated as 50% or higher, mainly in tonsils and base of tongue cancers [1]. The major focus in prevention is however on elimination of the tobacco smoking and alcohol consumption [2]. For example, during 2018 in Europe 3.1% of new HNSCC patients were diagnosed where a higher prevalence was observed for males and an estimated death rate among all cancer cases was 2.8% [3]. Globally, HNSCC accounts for more than 550,000 cases annually [4]. The treatment efficiency in terms of 5-year survival rate is quite high if the cancer is detected at an early stage. The usual therapeutic approach for HNSCC includes chemotherapy, surgery for cases that are operable, and radiation therapy. Surgery with or without radiation therapy is considered as the primary treatment option whereas chemotherapy is usually prescribed as an adjuvant or supplementary treatment. Despite the rather good success of therapy in early diagnosed cases, the mortality rate remains unchanged and is especially a problem in advanced cases or cases with metastases [5,6]. Targeted therapies of HNSCC, for example epidermal growth factor receptor (EGFR) kinase inhibitors or monoclonal antibodies directed on PD-1 (programmed cell death-1) or vascular endothelial growth factor (VEGF), have been tested in such cases and represent an additional option in tumor spread control. Nevertheless, these targeted therapy options require additional studies [7,8]. A study was also conducted on a potential correlation between programmed death ligand 1 (PD-L1) expression and prognosis of patients with oral squamous cell carcinoma (OSCC). No relation between PD-L1 expression and overall survival, disease free survival, and disease specific survival was found [9]. Considering the fact that the molecular basis of HNSCC is rather complex and the intra-tumor heterogeneity is extraordinarily high, targeted therapy will probably yield limited solutions. Interestingly, a more systemic approach towards understanding of the HNSCC pathogenesis has been recently acknowledged and it focuses on abnormalities in the functioning of the circadian system in humans. The circadian system impairment has been indeed, identified in diverse types of cancers including breast, colorectal and prostate cancer [10]. The biological clock, i.e., the circadian clock and the cell cycle are closely interconnected and changes or impairments in this regulatory network that shares common molecular elements are highly relevant for the tumor growth and cancer cells’ proliferation deregulation. The clock genes therefore, may have a big influence on the process of oncogenesis [11]. We propose that further investigation and understanding of clock gene involvement, and circadian clock regulation, in tumor development might contribute to novel insights into cancer pathogenesis.

## 2. Understanding the Head and Neck Squamous Cell Carcinoma at Molecular Level

The pathogenesis of head and neck cancers, including HNSCC, is a complex process when genetic mutations coupled with altered protein expression change the cell microenvironment, which is then supportive for uncontrolled cell growth and tumor development (Table 1). Some of the best studied molecular events in HNSCC are alterations in the tumor-suppressor and apoptosis induction gene *p53*, with mutations detected in over 50% of HNSCC cancers [12,13] and in the tumor-suppressor *PTEN* gene as well as corresponding regulatory pathways. Interestingly, *NPAS2,* one of the main circadian clock genes involved in DNA damage response mechanisms, has also been considered as a tumor suppressor gene and its alterations were already correlated with the tumor stage and metastases occurrence [14,15]. Moreover, it has been reported that circadian clock influences both intrinsic as well as extrinsic apoptosis pathways. In addition, circadian clock might affect cancer treatment efficiency through modulation of the pharmacokinetics and pharmacodynamics of chemotherapeutic medications along with the DNA repair enzymes activity responsible for repairing DNA damage caused by anticancer drugs [16,17]. In addition, it has been found that in HPV-positive HNSCC, *PIK3CA* mutations that seem to cooperate with HPV oncoproteins E6 and E7, are also present at a higher rate. Recent studies of the genetic alterations in HNSCC also revealed mutations of the *NOTCH1* gene and other NOTCH family members [18]. These genes and corresponding pathways are important in regulation of the cell cycle and cell differentiation that are clearly disrupted in malignant processes. Particularly, the NOTCH1 and PIK3CA pathways are impaired in HNSCC which has clinical implications and have thus, been studied as possible targets in clinical trials. The PI3K pathway has been shown to regulate numerous cellular processes such as the programmed cell death (apoptosis), proliferation, cell cycle progression, cytoskeletal stability and motility, and energy metabolism [19,20]. The activation of this pathway induces enhanced expression of numerous proliferative and anti-apoptotic proteins and the pathway has been found to be activated in up to 50% to 80% of HNSCC [21]. Importantly, the PI3K pathway may be activated through the EGFR which was also acknowledged as a possible target for tyrosine kinase inhibitors in HNSCC [22]. Still, it seems that clinical outcomes with tyrosine kinase inhibitors Erlotinib and Gefitinib were not satisfactory so far. In addition, in combined regimens of the EGFR directed monoclonal antibody Cetuximab with chemo- or radiotherapy with a satisfactory result, no correlation of the therapeutic efficacy was observed with EGFR copy number, expression or mutations [23]. The cellular signaling pathways of the receptors Janus kinase (JAK) activators of the transcription factor STAT and phospholipase-Cy/protein kinase C, are also activated along with EGFR phosphorylation [24]. Similarly, most epithelial carcinomas overexpress and constitute functional activation of the EGFR family or receptors [25]. For instance, diurnal suppression of EGFR signaling by glucocorticoids was reported in mice, therefore the treatment of EGFR-driven tumors in animals with specific kinase inhibitor was more effective when it was given during the resting period of the day, while glucocorticoids are low. These evidences corroborate that circadian clock has to be taken under consideration in cancer treatment [26].

All these molecular events observed in HNSCC are manifested as impaired cell growth regulation, increased tumor proliferation, migration and angiogenesis that contribute to the carcinogenic transformation. It has been postulated that many of the observed DNA alterations in tumor cells are due to oxidative reactions that induce DNA damage [27]. In the HNSCC pathogenesis, numerous chemical compounds may be correlated with this process, such as those substances present in tobacco. Apart from it, benzo[a]pyrene diol epoxide, a known carcinogen found in tobacco also induces genetic damage, including those of the *P53* gene [28]. The DNA damage repair mechanisms, including the double or single strand break repair mechanisms have been found to be altered in the HNSCC as well. Furthermore, different polymorphisms have been linked with DNA repair mechanisms alterations in HNSCC even though this depends primarily on the race and risk factors, such as for example tobacco smoking [29]. Some DNA repair mechanisms are under direct control of the circadian clock, namely the nucleotide excision repair [16]. Recently the *xeroderma pigmentosum* group A (XPA) protein, representing the rate-limiting subunit of excision repair, essential for the body’s DNA damage repair mechanism, was for example found to be under circadian regulation as well [30].

## 3. The Fundamental Physiology of Biological Clock

Circadian is an internal timekeeping system that evolved in organisms through the evolutionary process over a period of millions of years allowing organisms to cope and accommodate with the daily cycle of light and darkness. It is referred as “body clock” which is an inbuilt and nearly automatic rhythm, which repeats itself at about 24-h interval. The suprachiasmatic nuclei (SCN) neurons have a near-24-h rhythm of electrical activity, even in the absence of environmental signals. All organisms have the internal biological clock that controls several important physiological functions for instance hunger, sleep and awake pattern, endocrine and metabolism control, gene expression etc., and all of them are closely entwined [41].

An important molecule within this mechanism is melatonin, a hormone produced in the brain at night whose level in the body follows a daily cycle. It is known that bright light suppresses melatonin production. The level of melatonin affects the production of other important hormones in the body, e.g., estrogen and serotonin. When light reaches the eye, it travels through the lens to the retina. At the retina rods allow vision at low light levels whilst cones distinguish colors, red, blue, and green. In combination they convert light into neurological signals, which the brain interprets and translates into images. Similarly, a receptor located in retina, has been found extremely sensitive to blue light and sends neurological signals that help our biological clock to function effectively [42]. The third receptor respond to light in a non-visual way, in contrast to the rods and cones, by sending signals to the central pacemaker, which is located in the SCN of the anterior hypothalamus in the brain. The clock located in SCN and daily light-dark cycle is entertained to 24-h a day initiating at the neurons of the retina and going to the SCN neural pathways [43,44].

The SCN is a pair of accumulated nerve cells lying in the hypothalamus, just above the optic chiasma. Each nucleus contains about 10,000 neurons. The physiological basis of the circadian rhythm lies in the interactions, based on negative feedback loop, between specific groups of proteins creating the “tick” of the biological clock.

### 3.1. Circadian Clock Genes-Fundamentals

**Figure 1:**
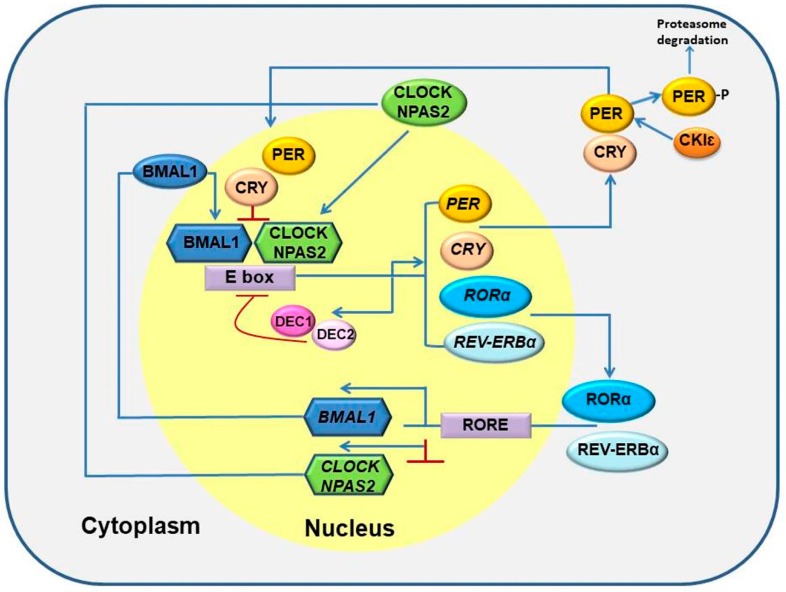
Schematic representation of circadian clock genes: The mechanism of core circadian clock depends upon a transcriptional-translational feedback loop comprising the transcription factors BMAL1 and CLOCK/NPAS2. The positively regulated gene *BMAL1* and *CLOCK/NPAS2* hetero-dimerize and bind E-box elements in *PER* and *CRY* genes. The PER and CRY proteins inhibit their own transcription by interfering with CLOCK/NPAS2 and BMAL1 activity. *DEC1* and *DEC2* are involved in the regulation of circadian genes as well. A second feedback loop involving opposing action of REV-ERBα and RORα. Additionally, CK1ε phosphorylates PER and tags it for proteasome-mediated degradation. Blue arrows represent activation, red lines represent suppression and thin blue arrow represents signal for proteasome degradation.

There are numerous genes involved, either directly or indirectly, in the entire system of circadian clock. The system is composed of two oscillators: the central (SCN) and the peripheral (localized in different organs in the body). The regulations of central and peripheral circadian oscillators involve transcriptional-translational feedback loops that consist of so-called core circadian clock genes such as *PERIOD* (*PER1*, *PER2* and *PER3*), *CRYPTOCHROME* (*CRY1* and *CRY2*), *CLOCK* (circadian locomotor output cycles kaput), *NPAS2* (neuronal PAS domain protein), *BMAL1* (brain and muscle ARNT-like protein 1, referred in nomenclature as ARNTL), retinoic acid-related orphan nuclear receptors *RORα* and *REV-ERBα*, *CASEIN KINASE 1є* (*CK1є*), and *TIMELESS* (*TIM*) [41,43,45,46]. The entire loop system depends upon genes that are both positive regulators, such as bHLH-PAS (basic helix-loop-helix-PAS) transcription factors CLOCK/NPAS2 and BMAL1, or negative regulators such as PER, CRY, and TIM in oscillators. The CLOCK/NPAS2 and BMAL1 heterodimerize and stimulate the expression of the three *PER* genes and the two *CRY* genes as well as *RORα* and *REV-ERBα* genes by binding to an E-box enhancer region in their promoters (Figure 1). In the cytoplasm of those neurons, in the direction of *PER* and *CRY* genes, proteins are used to form PER-CRY complexes and subsequently taken into the nucleus to suppress the BMAL1 and CLOCK/NPAS2 mediated transcription. In the second loop RORα and REV-ERBα regulate the expression of *BMAL1* and presumably *CLOCK/NPAS2* genes by competing for the ROR elements in their promoter regions, whereas RORα up-regulates and REV-ERBα down-regulates *BMAL1* and *CLOCK/NPAS2* expression. Thus, the noticeable feature of the entire circadian system is not only the circadian clock gene expression, but also the downstream circadian clock-controlled genes’ mRNA and corresponding protein expression [5,47,48]. Additionally, majority of the clock proteins are post translationally modified by number of kinases and phosphatases, crucial for sustainment of circadian rhythmicity [48].

Numerous epidemiological studies, done on employees working night shifts, have shown that the disruption in circadian rhythm has an association with cancer development and that is particularly true for breast cancer, skin cancer, colon cancer, oral cancer etc. [49,50,51,52,53,54]. On the other hand, studies also demonstrate that hepatocellular carcinoma, chronic myeloid leukemia (CML), HNSCC etc., are also associated with disrupted expression of circadian clock genes [5,55,56,57].

However, little is known about the circadian control of HNSCC and a clear confirmation of association and the functional roles of circadian clock genes in HNSCC is still under investigation.

### 3.2. Circadian Genes and the Cell Cycle

**Figure 2:**
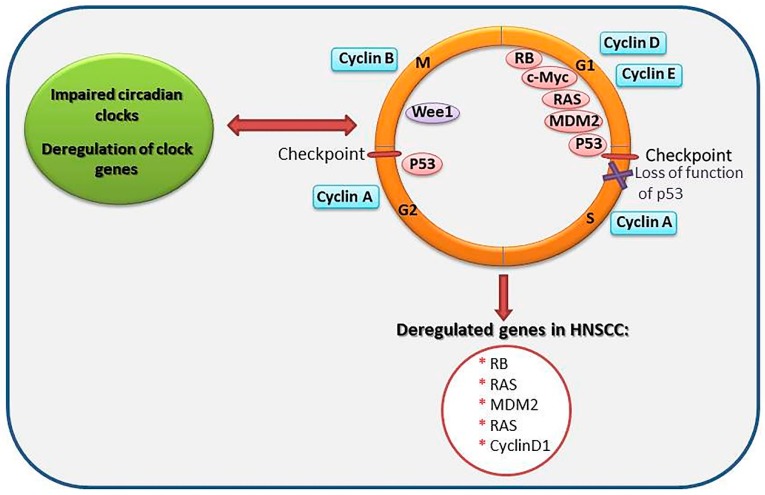
Schematic representation of circadian clock genes association with the cell cycle. Mutations in the p53 gene may be a selective feature for cancer cells, permitting them to intrude various cell cycle checkpoints and to avoid apoptosis and senescence. Cancer cells, therefore, proliferate under specific conditions not inherent to normal cells. The asterisks marks some of previously described molecules that are deregulated in head and neck squamous cell carcinomas (HNSCC) and that are involved in the cell cycle regulation. The red inverted arrow represents impaired circadian clocks lead to disruption of cell cycle and cell cycle disorder also affect circadian clocks. The red arrow represents the disruption of cell cycle leads to deregulation of genes in HNSCC. Red ellipse stands for checkpoints in the cell cycle. The purple multiple sign displays loss of function of the p53 in the cell cycle.

It is widely believed that the expression of about 2–10% of all mammalian genes are under the control of clock genes. This phenomenon is primary tissue- or organ-specific; however, a few genes, under the control of circadian clock and encoding the process of cell cycle progression, are expressed in multiple organs [44]. Those genes participate in the regulation of circadian clock and the cell cycle/DNA damage checkpoints and are inter-linked both functionally and at the molecular level, apparently becoming pivotal in maintaining the integrity and stability of the genome [58]. As a matter of fact, the two regulatory mechanisms; the cell cycle and the circadian rhythm affect directly or indirectly all biochemical reactions in the body. Therefore, any disruption in these regulatory mechanisms may have fatal consequences for the cell [59]. It has been well documented that in dividing cells, major circadian clock components affect the cell cycle by regulating *Wee1* expression, a kinase that regulates Cdc2 activities and hence the transition from G2- to M-phase of cell cycle [60]. Similarly, another study concluded that mice with *Per2* mutation possessed constitutively high levels of *c-Myc* expression, a cell growth/proliferation gene, and diminished *p53* gene expression, the latter bearing an unprecedented role in the regulation of the cell cycle G1-S checkpoint [61] (Figure 2).

### 3.3. Association between Deregulation of Circadian Genes During Transcription and Other Illnesses

About 10% of genes, which are known as the so called ‘clock-controlled genes’, remain under the control of the circadian clock [62,63]. Many of them are tissue specific and their genetic expressions vary among organs and systems of the body [64,65,66,67]. Deregulation of the clock components CLOCK and BMAL1 have been suggested to cause serious metabolic disorders resulting in serious disorders, such as for example diabetes mellitus and decreased insulin synthesis [68].

Considering that the mechanism of the clock regulation through transcription is quite complex and technically demanding, the investigation of gene expression patterns might be quite helpful. For example, lower levels of *CLOCK* were observed in tumor-unaffected tissues in comparison to the affected tissues, in patients with breast cancers that indicates an aberrant overexpression of *CLOCK* as a possible early event in carcinogenesis [69]. Still, a careful approach towards phenotypes’ studies that result from circadian genes’ disruption is needed as changes in gene expression might be due to genetic expression, loss of rhythmicity or disturbances in specific effects [70].

In addition, epigenetic factors should be also taken into account while transcriptional regulation of clock genes is analyzed. For example, Taniguchi et el. showed that the *BMAL1* gene was transcriptionally silenced by promotor CpG island hyper methylation in hematological malignancies, where it might hamper development of the physiologic circadian rhythm [71]. Epigenetic regulation of gene expression is central both to physiological regulation of gene expression and development of cancers. For instance, the hallmarks of cancer include evasion of apoptosis, autocrine growth regulation, resistance of antigrowth signals, sustained angiogenesis, limitless replicative potential, invasiveness and ability to forming metastasis [72].

## 4. Relation between Circadian Clock Gene and Cancer Development

Disruption in circadian rhythms is associated with several forms of human cancers. An increasing dysfunction of the clock work has already been attributed to the pathogenesis of cancer and many studies revealed that circadian clock gene deregulation is involved in the development of many diseases including malignancies. Among studied malignancies in correlation with the circadian clock impairment, breast cancer has been studied in more details. Few studies revealed that decreased expression levels of *PER* genes have been observed in tumor tissues compared to the normal adjacent tissues in cancers of various body parts [54,73,74,75,76,77,78,79]. Moreover, a correlation between tumor metastasis and life expectancy in terms of prognosis of breast cancer patients has clearly been underlined by *NPAS2* [73,80,81]. Indeed, *NPAS2* has already been established as a prognostic biological marker in breast cancer as well as in colorectal cancer [80,82]. Cell propagation and invasion are associated with lower *NPAS2* expression that eventually leads to increased wound healing ability in colorectal cancer cells, indicating the pivotal role of *NPAS2* in tumor inhibition [74].

On the other hand, deregulation of *BMAL1* gene has been associated with various cancers such as breast, colorectal, prostate, pancreatic, ovarian cancers, HNSCC, B-cell lymphoma, pleural mesothelioma, acute lymphocytic leukemia, acute myeloid leukemia, and CML [56,71,75,82,83,84,85]. For example, multiple studies have revealed lower expression of *BMAL1* in tumor tissue compared to healthy adjacent tissues in patients with pancreatic cancer and pancreatic ductal adenocarcinoma, as well as colorectal cancers [75,86,87]. Furthermore, lower expression levels of core circadian *CRY2* gene in breast cancer tissues was associated with breast cancer advancement and the disease outcomes [88]. A report shows that the combination of low *CRY1* and low *BMAL1* expression were prognostic factors in ovarian cancers, however, neither *CRY1* nor *BMAL1* were linked with the survival rates of patients suffering from ovarian cancers [89]. The use of estrogen hormone also had an association with *CLOCK* gene expression and development of breast cancer [90] and in colorectal cancer, increased expression of *CLOCK* has been reported in tumor tissue compared to healthy adjacent tissues [91]. Moreover, employing a synthetic REV-ERBα/β agonist, a diminished cell proliferation of the breast cancer cell lines occurred, and this was not dependent on the estrogen receptor or HER2 status of the cell lines. It was therefore, proposed that the most effective method for treating breast cancer might be also through targeting of the REV-ERB [92]. Besides, several studies shown that DEC1 and DEC2 play a pivotal role in circadian rhythm regulation, cell growth, apoptosis etc. It is a fact that an enhanced or decreased expression of DEC1 and DEC2 may regulate tumorigenesis [93]. Collectively, those findings confirm the fundamental role of the circadian clock in tumor pathogenesis. Moreover, it has been demonstrated that a “repaired” circadian clock function in cancer cells might indeed, inhibit tumor growth. Therefore, triggering the biological clock in tumors might be a new approach for slowing down malignant progression, opening a niche for new opportunities and for the chronobiological intervention in cancer treatment protocols [94]. The concept of cancer chronotherapy is based on existence of endogenous biologic rhythms that can be exploited for design and optimization of the cancer treatment with the aim to improve the patient outcome [95]. Identifying the connections between impaired clockwork and cell cycle networks might shed light on cancer progression and may be used for improvement of cancer patients’ treatment outcomes. It might be therefore, envisaged that a chronotherapy-based approach to HNSCC treatment might include delivery of anticancer treatment at a proper time, i.e., at a time when a reduced toxicity towards healthy tissue is observed. Further investigation on asynchronicity of the circadian profile and cell cycle gene activity in HNSCC and healthy tissue may thus provide important information for such a chrono-therapeutic approach. For example, the study by Tsuchiya at al. on the influence of a dosing-time of docetaxel-, cisplatin- and 5-fluorouracile-induced toxicity in patients with OSCC, already demonstrated that a chronotherapy regimen (evening-dosing) might reduce severe adverse effects of these drugs in clinical practice [96]. However, large interpatient differences in circadian functions should be taken into account, and a significant variability in response to chronotherapy is expected. These speaks in favor of development of patient-tailored, personalized chronotherapies where an interdisciplinary systemic approaches are needed to combine mathematical modeling based on cellular and whole-body physiology with preclinical and clinical data [95].

### 4.1. Association between Circadian Clock Genes and Head and Neck Squamous Cell Carcinoma

Even though technological and research progress have been made in the arena of cancer treatment, the mortality rate is quite high and hence the quest for new treatment approaches, proper diagnostic tools and biomarkers, is essential. A growing body of evidence connects circadian deregulation with cancer development, but very little is known so far about HNSCC and aberrant circadian clock. Hsu et al. were the first to provide evidence linking the development of HNSCC with disturbances of circadian clock genes expression [5]. Hsu et al. studied the expression of nine circadian clock genes *PER1, PER2*, *PER3*, *CRY1*, *CRY2*, *CKIε*, *TIM*, *CLOCK* and *BMAL1* in tumor and non-tumor, adjacent tissue from 40 patients diagnosed with HNSCC. Findings of studies show that the expression level of *PER* genes, *CRY1* and *BMAL1* genes were significantly lower in HNSCC [5]. Of the seven-downregulated genes studied, the *CRY2* was the most downregulated gene, a nine times decrease in tumor tissue compared to the non-tumor tissue was measured. Furthermore, it has also been established that downregulated *PER3*, *CRY2* and *BMAL1* was attributed to the more advanced stages of cancer.

Moreover, analysis of 40 HNSCC patients’ pathological reports, were done to examine the correlation of the circadian clock genes expression with HNSCC tumor-dependent variations. In the process, the patients were divided into two groups: one group with tumor size <3 cm and tumor depth <1 cm, and the other group with tumor size >3 cm and tumor depth >1 cm. Analysis indicated that amongst nine studied clock genes, transcripts of *PER3* highlighted a tumor size-dependent variation and an invasive depth-dependent variable pattern. On the other hand, *TIM* displayed only a tumor size- dependent variation pattern. Furthermore, a bigger size of tumor was related with diminished *PER3* and enhanced *TIM* expression, and increased tumor invasion was associated with decreased *PER3* expression [5]. Analysis of the association between survival rates of the patients with altered circadian clock gene expression have been done with a follow-up of 2 years post-surgery. Prospective analysis was done asses any link between patient’s survival status and circadian clock gene expression. It has been found that downregulation of *PER1* and *PER3* were correlated with poor survival rates [5].

In the same study, altered circadian clock gene expression was analyzed in the context of age differences and the patients were further stratified into young (30–45 years old), middle-age (45–60 years old) and old-age (60–80 years old) groups. It was found that the *CK1є* was significantly downregulated in the middle-age patient group in comparison with the young patient group. Expression of *TIM* was also significantly impaired in the middle-aged patient group in comparison with young and old- age groups. The authors assumed that in HNSCC, downregulated and disrupted circadian clock genes probably lost their function in removing pre-malignant and malignant cells leading to appearance of malignancies [5]. In addition, it is probable that deregulation of circadian machinery influences the molecules involved in cell cycle progression and apoptosis regulation, thus leading to cancer progression. Hence, it has been hypothesized that circadian clock genes can regulate or suppress other circadian genes by certain mechanisms, which may also corroborate the finding on simultaneous lower expression levels of circadian gene such as *PER1*, *PER2*, *PER3*, *CRY1*, *CRY2* and *BMAL1* in HNSCC (Table 2) [5].

Hsu et al. further reported on relationship between the circadian clock gene expression and severity of disease, tumor size, tumor invasion, survival rate. They found that various altered genes were associated with different clinical parameters. *PER3* was the only gene correlated with all investigated parameters, though more profound investigations are required to elucidate the causes of *PER3* deregulation in HNSCC in particular [59]. The authors also investigated alterations of nine clock genes, namely *PER1*, *PER2*, *PER3*, *CRY1*, *CRY2*, *CLOCK*, *CK1ε, BMAL1* and *TIM* in peripheral blood of HNSCC patients [97]. They found that all nine clock genes were downregulated and suggested the *PER1* and *CLOCK* genes as potential circulating prognostic markers for HNSCC. Interestingly, *CLOCK* gene was the most downregulated gene among studied, nine-downregulated clock genes with at least a three times decrease in patients in comparison with healthy individuals. In this study, tumor samples from both oral and non-oral areas were included. It was again observed that the same *CLOCK* gene was the most downregulated one. It should be highlighted that the degree of *CLOCK* gene expression was significantly different between oral and non-oral tumors. Importantly, the recovery of *PER1* and *CLOCK* expression in postoperative patients was correlated with good prognosis. The authors hypothesized that downregulation of *CLOCK* gene inhibited protective function of *NF-κB* activation [97].

Collectively, these data provide a good basis for pursuing investigations on the involvement of the highly complex circadian system and its core clock components in the malignant progression of HNSCC and other tumors as well.

### 4.2. Association between Circadian Clock Genes and Oral Squamous Cell Carcinoma

OSCC is predominantly a disease of elderly males who have a long history of smoking tobacco and/or drinking alcohol [103]. The most commonly affected areas in OSCC are the tongue followed by the floor of the mouth, gingiva and alveolar mucosa [104]. Furthermore, the American Joint Committee on Cancer (AJCC) re-evaluated the staging criteria and lymph node ratio (LNR) staging of their 7th edition in the 8th edition for staging of the tongue squamous cell carcinoma (TSCC) to improve identification of TSCC patients with poor prognosis. Tumor staging was indeed, revised on the basis of depth of invasion, extranodal extension, and LNR. Their findings show that among all reclassified patients, the majority of patients received an upstage in the staging score, while the others were classified in the same stage group as previously. Moreover, the patients receiving upstage in reclassification had more recurrences or an increased mortality rate. It has been therefore, suggested that the 8th edition of the AJCC criteria allows for better stratification of TSCC patients [105]. As discussed earlier, the clock genes have a clear role in cancer development, prognosis and therapy. It has been suggested that the *PER1* gene may be used as a marker to determine clinical staging and the metastatic risk. It can also be used as a novel target for the prevention and treatment of oral cancer [54,101,106,107].

Chen et al. investigated the *PER1* expression in 41 OSCC patients by comparing cancerous tissue with healthy adjacent mucosa and detected the correlation of *PER1* expression with clinico-pathological features in these patients. The result revealed a lower expression level of *PER1* in tumor tissue compared to the non-tumor, adjacent tissue and the expression level decreased with the tumor progression (Table 2) [101]. Apart from this, recent studies demonstrated that *PER2* expression was reduced in OSCC compared with adjacent, non-tumor tissue. Further analyses shown that the expression of *PER2* was associated with the clinical stage of OSCC, the lymphatic metastasis status and the patient survival time. Reduced expression of *PER2* seems therefore, to promote OSCC and shorten survival time [102]. It has also been found that downregulation of *PER2* in OSCC cells may reduce cell apoptosis [108,109]. Another important apoptosis-related gene is caspase-8, and its abnormal expression was found to be associated with the carcinogenesis process [110]. Previous studies have already demonstrated decreased caspase-8 expression in various types of cancers [111].

Furthermore, Bjarnason et al. shown that various clock genes, in the oral mucosa of healthy diurnally active volunteers, are under circadian regulation [112]. Their study provided the initial evidence of rhythmic circadian expression profile of *PER1*, *CRY1*, and *BMAL1* (early in the morning, in the late afternoon, and at the night respectively) in oral mucosa of humans, alike to those found in the SCN and the peripheral tissues in rodents [112]. Additionally, the major peak in *PER1* expression in oral mucosa matched with the G1-phase marker (*p53*) of the cell cycle, while the peak for *BMAL1* coincided with the M-phase marker *cyclin β1*, suggesting a potential functional correlation between the circadian clock and the mammalian cell cycle, in support of a circadian coordination of cell-cycle events in oral mucosa [112].

On the other hand, Zhao et al. demonstrated that *PER1* expression was significantly diminished in tumor tissues compared to the adjacent, non-tumor tissue of the patients with buccal squamous cell carcinoma (BSCC) [98]. Further analysis, using cutting-edge technologies showed decreased *PER1* expression correlated with the advanced clinical stages and increased risk of metastasis to the regional lymph node. The MMP-2 (matrix metallopeptidase 2) playing an important role in tumor cell invasion and metastasis, had an oppositely correlated expression to *PER1*. This means, that the expression of MMP-2 was increased as *PER1* expression was decreased. The study suggests that down-regulated *PER1* expression is correlated with more advanced cancer stages in patients with BSCC. This corroborates an anti-oncogenic role of *PER1* whose expression may correlate with invasion and metastasis of BSCC cells. The *PER1* expression in BSCC and its association with the patients’ clinico-pathological parameters indicate that *PER1* expression might be used to evaluate the stage and metastatic risk of patients with BSCC [98].

Similarly, Deng et al. examined the expression level of circadian clock genes (*PER1*, *PER2*) to explore their correlation with VEGF. Their result showed that the expression of clock genes *PER1* and *PER2* were decreased significantly in esophageal squamous cell carcinoma (ESCC) tumors with different proliferation, differentiation and different TNM stage [100]. Moreover, the expression levels of *PER1* and *PER2* had a negative association with lymph node metastasis, distant metastasis and clinical staging. Therefore, *PER1* and *PER2* exhibited a suppressive effect on the progression and migration of ESCC. A significant negative correlation was also found between *PER1/PER2* activity and VEGF expression in ESCC patients’ cancer tissue, showing that decreased levels of *PER1*/*PER2* may influence VEGF levels [100].

To get some new insights into the mechanism by which the circadian system affects tumorigenic process and so the treatment outcome, Tang et al. examined the levels of *BMAL1* and the rhythmic patterns in TSCC. Both were affected in TSCC clinical samples and tested cell lines. The level of *BMAL1* was decreased notably in TSCC and as well as in the adjacent non-tumor tissue compared with normal tongue epithelial tissue [99].

## 5. Conclusions

The circadian clock malfunctioning has been found as one underlying process in many pathologies, including malignant diseases. The core protein synthesis that is under control of clock genes and corresponding transcription and translation mechanisms, may influence directly or indirectly the activity of proteins involved in the cell cycle progression but also in the DNA repair mechanisms. Findings presented in the scientific literature support indeed, a possible correlation between circadian clock gene expression with malignant diseases’ pathogenesis, including HNSCC as well. While this correlation is rather obvious, a clear mechanism that can be exploited in the management of malignant disease or the development of any new therapeutic strategy, is still lacking. More research into this field would, therefore, probably yield some new and interesting data to be exploited in a clinical environment. In particular, studies on animal models or in silico analyses of molecular pathways may be required before any advancements may be achieved in diagnosis, therapeutic and prognostic strategies of HNSCC based on circadian clock. Interestingly, some of the known bioactive compounds have already been found to directly influence the circadian period. One of such compounds is the dehydroepiandrosterone that may reduce periodicity of circadian rhythm. More importantly, numerous tyrosine kinase inhibitors may also act on the clock function [113]. A systemic approach towards HNSCC and other malignant disease management and treatment may, therefore, be useful for improved disease management in the future.

## Figures and Tables

**Table 1:** A list of genes with a role in head and neck squamous cell carcinomas (HNSCC).

Gene	Symbol	References
CyclinD1	CyclinD1	[31]
Tumor protein p53	p53	[31]
Phosphatidylinositol-4,5-Biphosphate 3-Kinase catalytic Subunit Alpha	PIK3CA	[32]
Cyclin-dependent kinase inhibitor 2A	CDKN2A	[33]
Neurogenic locus notch homolog protein 1	NOTCH1	[34]
Protocadherin Fat 1	FAT1	[33]
Monocyte to macrophage differentiation-associated protein 2	MMD2	[35]
Retinoblastoma 1	RB1	[33]
Epidermal growth factor receptor	EGFR	[36]
Tyrosine-protein kinase Met	MET	[37]
Lysine methyltransferase 2D	MLL2	[33]
Nuclear receptor binding SET Domain Protein 1	NSD1	[33]
HRas proto-oncogene	HRAS	[38]
Phosphatase and tensin homolog	PTEN	[39]
Transforming Growth Factor Beta Receptor 2	TGFBR2	[40]

**Table 2:** Association of circadian clock genes in HNSCC.

Cancer Type	Deregulated Clock Gene	Result	Ref.
HNSCC	*PER1, PER2, PER3, CRY1, CRY2, BMAL1, CLOCK* *TIM*	Downregulation Upregulation	[5,97]
BSCC	*PER1*	Downregulation	[98]
TSCC	*BMAL1*	Downregulation	[99]
ESCC	*PER1*	Downregulation	[100]
OSCC	*PER1, PER2*	Downregulation	[101,102]

## Abbreviations

HNSCC	Head and neck squamous cell carcinoma
SCN	Suprachiasmatic nuclei
CML	Chronic myeloid leukemia
OSCC	Oral squamous Cell Carcinoma
TSCC	Tongue Squamous Cell Carcinoma
BSCC	Buccal Squamous Cell Carcinoma
ESCC	Esophageal Squamous Cell Carcinoma
VEGF	Vascular Endothelial Growth Factor
EGFR	Endothelial growth factor receptor
